# Exploring the role of drug-metabolising enzymes in antidepressant side effects

**DOI:** 10.1007/s00213-015-3898-x

**Published:** 2015-03-12

**Authors:** Karen Hodgson, Katherine E. Tansey, Rudolf Uher, Mojca Zvezdana Dernovšek, Ole Mors, Joanna Hauser, Daniel Souery, Wolfgang Maier, Neven Henigsberg, Marcella Rietschel, Anna Placentino, Ian W. Craig, Katherine J. Aitchison, Anne E. Farmer, Richard J. B. Dobson, Peter McGuffin

**Affiliations:** 1 MRC Social, Genetic and Developmental Psychiatry Centre, Institute of Psychiatry, Psychology and Neuroscience, King’s College London, London, UK; 2 Institute of Psychiatry, Psychology and Neuroscience, King’s College London, London, UK; 3 Department of Psychiatry, Dalhousie University, Halifax, NS Canada; 4 University Psychiatric Clinic, Ljubljana, Slovenia; 5 Research Department P, Aarhus University Hospital, Risskov, Denmark; 6 Laboratory of Psychiatric Genetics, Department of Psychiatry, Poznan University of Medical Sciences, Poznań, Poland; 7 Laboratoire de Psychologie Médicale, Université Libre de Bruxelles; PsyPluriel - Centre Européan de Psychologie Médicale, Brussels, Belgium; 8 Department of Psychiatry, University of Bonn, Bonn, Germany; 9 Croatian Institute for Brain Research, Medical School, University of Zagreb, Zagreb, Croatia; 10 Division of Genetic Epidemiology in Psychiatry, Central Institute of Mental Health, Mannheim, Germany; 11 Psychiatric Unit (UOP 23), Department of Mental Health, Biological Psychiatry Unit Spedali Civili Hospital of Brescia, IRCCS-FBF, Brescia, Italy; 12 Department of Psychiatry, University of Alberta, Edmonton, AB Canada; 13 NIHR Biomedical Research Centre for Mental Health and Biomedical Research Unit for Dementia at South London and Maudsley NHS Foundation, London, UK; 14 The Lundbeck Foundation Initiative for Integrative Psychiatric Research, Aarhus, Denmark

**Keywords:** Antidepressant, Pharmacogenetics, Side effects, Drug metabolism, Cytochrome P450 enzymes

## Abstract

**Rationale:**

Cytochrome P450 enzymes are important in the metabolism of antidepressants. The highly polymorphic nature of these enzymes has been linked to variability in antidepressant metabolism rates, leading to hope regarding the use of P450 genotyping to guide treatment. However, evidence that P450 genotypic differences underlie the variation in treatment outcomes is inconclusive.

**Objectives:**

We explored the links between both P450 genotype and serum concentrations of antidepressant with antidepressant side effects, using data from the Genome-Based Therapeutic Drugs for Depression Project (GENDEP), which is a large (*n* = 868), pharmacogenetic study of depressed individuals treated with escitalopram or nortriptyline.

**Methods:**

Patients were genotyped for the enzymes CYP2C19 and CYP2D6, and serum concentrations of both antidepressant and primary metabolite were measured after 8 weeks of treatment. Side effects were assessed weekly. We investigated associations between P450 genotypes, serum concentrations of antidepressants and side effects, as well as the relationship between P450 genotype and study discontinuation.

**Results:**

P450 genotype did not predict total side effect burden (nortriptyline: *n* = 251, *p* = 0.5638, *β* = −0.133, standard error (SE) = 0.229; escitalopram: *n* = 340, *p* = 0.9627, *β* = −0.004, SE = 0.085), study discontinuation (nortriptyline *n* = 284, hazard ratio (HR) = 1.300, *p* = 0.174; escitalopram *n* = 376, HR = 0.870, *p* = 0.118) or specific side effects. Serum concentrations of antidepressant were only related to a minority of the specific side effects measured: dry mouth, dizziness and diarrhoea.

**Conclusions:**

In this sample where antidepressant dosage is titrated using clinical judgement, P450 genotypes do not explain differences between patients in side effects with antidepressants. Serum drug concentrations appear to only explain variability in the occurrence of a minority of specific side effects.

**Electronic supplementary material:**

The online version of this article (doi:10.1007/s00213-015-3898-x) contains supplementary material, which is available to authorized users.

## Introduction

Cytochrome P450 enzymes play a key role in the metabolism of antidepressant drugs into less active compounds. But drug metabolism rates vary between individuals, resulting in differences between patients in terms of antidepressant serum concentrations (Reis et al. 2009).

It has been shown that one of the causes of this variability in rates of antidepressant metabolism are the genotypic differences seen in the cytochrome P450 (P450) enzymes (Dahl et al. 1996; Grasmäder et al. 2004; Hodgson et al. 2014; Huezo-Diaz et al. 2012; Murphy et al. 2001; Rudberg et al. 2008). Kirchheiner et al. used this evidence to suggest genotype-based adjustments to dosage that would counter these differences (Kirchheiner et al. 2001; Kirchheiner et al. 2004).

It has been proposed that the variation in drug metabolism rates, and resultant variation in serum concentrations of antidepressant might play a role in whether or not antidepressant treatment is successful (Ingelman-Sundberg 2004) and go some way to account for the variability that is seen in outcomes between patients who are treated with antidepressants (Trivedi et al. 2006).

In particular, low rates of drug metabolism will lead to higher levels of antidepressant, and this could potentially explain why some individuals report high levels of antidepressant side effects. Side effects play an important role in clinical decision-making when prescribing antidepressants; and adverse drug reactions are frequently cited by patients as a reason for discontinuing antidepressant treatment (Bull et al. 2002). As would be expected, inadequate antidepressant treatment results in increased levels of relapse (Kennedy et al. 2002) as well as increased health costs (Masand, 2003). Therefore, it is important to try and understand the causes of antidepressant side effects and treatment discontinuation.

Additionally, there has been interest in the potential use of P450 genotyping to personalize antidepressant treatment, particularly in light of FDA approval of the AmpliChip CYP450 Test (de Leon 2006). However, a systematic review in 2007 indicated that there was little evidence to support clinical utility of P450 genotyping in depression, with a number of studies being underpowered (Matchar et al. 2007).

More recently, two reports have been published examining P450 genetic prediction of response and tolerance to antidepressant treatment in the large US-based multicentre Sequenced Treatment Alternatives to Relieve Depression (STAR*D) study. Whilst one report found no evidence of association with either outcome amongst patients taking citalopram (Peters et al. 2008), a more recent paper based on the same data (but limiting the analysis to non-Hispanic Caucasians) concluded that there was evidence of a link between P450 genotype and both tolerance and remission (Mrazek et al. 2011).

In Genome-Based Therapeutic Drugs for Depression (GENDEP; a European pharmacogenetic study where patients received either nortriptyline or escitalopram), we found no evidence for a relationship between treatment response and P450 genotype (Hodgson et al. 2014). In light of the potential impact of adverse drug reactions in determining treatment outcomes (Bull et al. 2002), in this paper, we wish to extend this work to also examine the potential links between rates of drug metabolism and antidepressant side effects.

A range of different adverse drug reactions (ADRs) are observed with antidepressant medications, each with differing pharmacological underpinnings. Therefore, we considered both a measure of overall side effect burden, as well as the relationship between drug metabolism and each specific ADR. Additionally, we looked at drug metabolism rates in two ways: firstly, using genotypic information from the P450 enzymes of relevance, and secondly, using serum concentrations of drug, which allow us to capture the combined effects of both genetic and environmental influences on drug metabolism rates. Finally, we explored the association between P450 genotype and study drop out, as an additional measure of treatment tolerability.

## Methods

### Sample

The GENDEP (Genome-Based Therapeutic Drugs for Depression) project is a European, multicentre, open-label antidepressant pharmacogenetic project. The complete GENDEP cohort (Tansey et al. 2012; Uher et al. 2009b) included 868 treatment seeking adults (19–72 years, 63 % females) diagnosed with moderate to severe unipolar depression, established using the Schedules for Clinical Assessment in Neuropsychiatry (SCAN) interview (Wing et al. 1998). Exclusion criteria were personal or family history of bipolar disorder or schizophrenia and/or active substance dependence. To minimize population stratification, all patients were of White European ancestry.

We randomly allocated patients with no contraindications to receive either nortriptyline (50–150 mg daily) or escitalopram (10–30 mg daily). A protocol-driven flexible dosing scheme was used. Nortriptyline is a tricyclic antidepressant with predominantly noradrenergic action, whilst escitalopram is a highly selective serotonin reuptake inhibitor. Patients with contraindications for one drug were offered the other antidepressant. We measured the severity of depressive symptoms weekly, with the Montgomery-Åsberg Depression Rating Scale (MADRS; Montgomery and Asberg 1979). Prior to treatment, mean scores on the MADRS were 28.76 (SD = 6.78). GENDEP was approved by ethics boards in all participating centres and all participants provided written consent after the procedures were explained. GENDEP is registered at EudraCT (no. 2004-001723-38, http://eudract.emea.europa.eu) and ISRCTN (no. 03693000, http://www.controlled-trials.com).

### Side effect measurements

Side effects were measured on a weekly basis using the self-report Antidepressant Side Effect Checklist (ASEC; Supplementary Materials), which has been shown to correlate well with the interview-rated UKU (Lingjaerde, Ahlfors et al. 1987; Uher et al. 2009a). The ASEC was also administered prior to treatment, to capture any pre-existing symptoms.

The ASEC measures a total of 21 side effects. Although each item on the ASEC is rated on a 4-point scale (0, absent; 1, mild; 2, moderate; 3, severe), moderate and severe ratings were uncommon (Uher et al. 2009a). Therefore, the 4-point scale was collapsed to give ratings of presence or absence of each ADR.

We considered side effects in two ways. Firstly, we calculated the total number of ADRs experienced each week, to give a measure of the overall side effect burden for the patient. Secondly, we examined the weekly presence or absence of each individual side effect separately, to investigate ADR-specific effects.

### Cytochrome P450 enzyme genotyping

Blood samples were buffered in an EDTA solution and a standard protocol was used for DNA extraction (Freeman et al. 2003). We genotyped all patients using the Roche AmpliChip P450 (Roche Molecular Diagnostics, Alameda, CA, USA), a micro-array that measures 33 variants in *CYP2D6* and 2 variants in *CYP2C19*; In addition, the common *17 allele observed within *CYP2C19* (Sim et al. 2006) was also genotyped, using a TaqMan SNP genotyping assay on the 7900HT sequence detection system (Applied Biosystems, CA, USA). Genotypes were determined using SDS software (Applied Biosystems). After genotyping, allelic variation was categorised using standard nomenclature for the P450 genes. We used four categories to classify *CYP2D6* genotypes: poor (PM), intermediate (IM), extensive (EM) or ultrarapid (UM) metabolisers (Rebsamen et al. 2009). For *CYP2C19*, we used six categories: poor (PM), intermediate (IM), intermediate plus (IM+), extensive (EM), extensive plus (EM+) or ultrarapid (UM) metabolisers (Mrazek et al. 2011).

We examined *CYP2C19* as the relevant P450 genotype in the escitalopram-specific analyses, with *CYP2D6* genotype included as a fixed effect covariate given the smaller reported role of the CYP2D6 enzyme (Olesen and Linnet 1999). We considered *CYP2D6* as the relevant P450 genotype in the nortriptyline-specific analysis. We also controlled for the use of relevant P450-inhibiting co-medications.

### Co-medication

There are a number of commonly prescribed medications which are known to act as inducers or inhibitors of the P450 enzymes. In GENDEP, each patient reported any medications taken that were additional to the prescribed antidepressant. We categorised these co-medications using the FDA classification of in vivo inhibitors or inducers (FDA 2011). In the week that we took the blood sample for the measurement of serum levels, 5.81 % of patients with serum concentration data reported taking a CYP2C19 and/or CYP2D6 inhibiting drug, but no patients reporting taking CYP2C19- and/or CYP2D6-inducing drugs. A list of these reported cytochrome P450-inhibiting co-medications can be found in Supplementary Materials.

### Serum measurements

After 8 weeks of antidepressant treatment, we drew blood from patients in order to measure serum concentrations of both the antidepressant taken (nortriptyline or escitalopram) and its primary metabolite (10-hydroxynortriptyline or desmethylcitalopram). By this point, steady state serum concentrations would have been achieved (Hiemke and Hartter 2000; Linder and Keck 1998). All serum analyses were performed at the Department of Clinical Biochemistry, Kings College Hospital, London (UK).

Details are published elsewhere (Hodgson et al. 2014), but briefly, escitalopram, desmethylcitalopram, nortriptyline and total 10-hydroxynortriptyline were measured using achiral turbulent flow liquid chromatography (Couchman 2012). Detection was by tandem mass spectrometry (MS/MS) (TSQ Vantage, Thermo Fisher Scientific, Hemel Hampstead, UK). The cis- and trans-isomers of 10-hydroxynortriptyline were resolved and assay calibration was based on the cis-isomer. Sample preparation in both cases was by protein precipitation. The method used was fully validated according to FDA guidelines (FDA 2001).

**Fig. 1:**
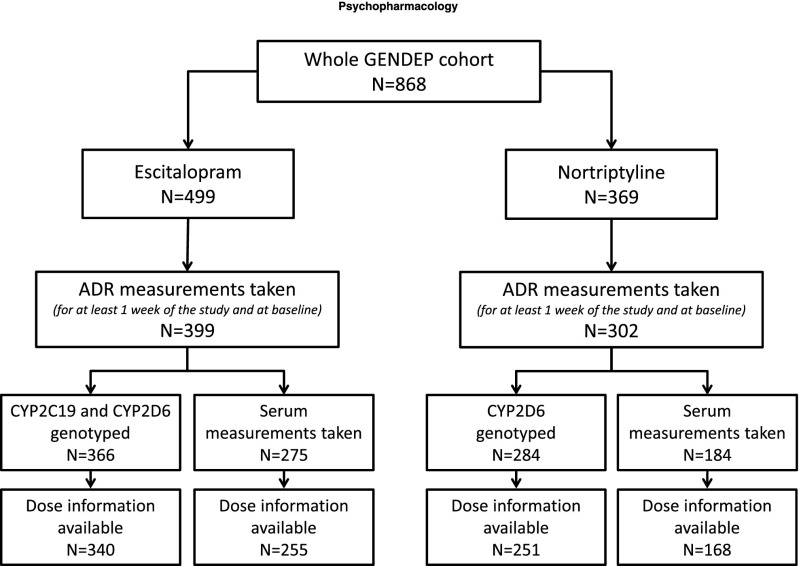
Diagram describing GENDEP sample

Four measures of serum concentration were considered in the study; concentration of drug, concentration of primary metabolite, ratio of metabolite to drug concentration, and total concentration of drug plus metabolite. Each of these serum measurements were standardised for ease of interpretation, with a mean of 0 and a standard deviation of 1. Figure 1 shows the number of patients where serum measurements and/or P450 genotypes were available.

### Statistical analysis

Given the different pathways for the metabolism of escitalopram and nortriptyline, all analyses were performed in a drug-specific manner. Previous analyses in this sample have established a relationship between P450 genotype and serum for both escitalopram and nortriptyline (more active forms of enzyme are associated with lower levels of antidepressant drug). The aim of this analysis was to consider how P450 genotype and serum concentrations of antidepressant might relate to the presence of ADRs.

As weekly measurements of ADRs were available, we used these repeated measures in our statistical model, with a Huber-White sandwich estimator of variance (Williams 2000), which provides standard errors that are robust to intra-individual correlations and so, relaxes the assumption of independent observations (Kent 1982).

Associations with total ADR burden were tested using linear models, whilst the weekly presence/absence of each specific ADR was examined using logistic models. When considering the specific ADRs, in order to correct for the 21 different outcomes, a Bonferroni correction for multiple hypothesis testing was applied; only associations where *p* < 0.002381 were considered significant (0.05/21 = 0.002381).

To ensure that reports of ADRs were not confounded by the severity of depression, MADRS scores were entered as a covariate in all analyses, along with baseline reports of ADRs, age, sex, linear and quadratic effects of time and centre of recruitment. When testing P450 genotype as a predictor, both P450-inhibiting medication and dose of antidepressant were used as covariates. Analyses of serum concentrations of antidepressants were performed in two stages. For the initial analysis of serum measurements, dose was not included as a covariate. We then performed secondary analyses with dose as a covariate for any ADRs which showed significant association with serum concentration of antidepressant, to probe the nature of the serum-side effect association.

Finally, P450 genotype was considered as a predictor of time to study discontinuation, using a survival Cox proportional hazards model. Covariates of age, sex, centre, baseline depression and baseline total ADR score were included in the model.

### Power calculations

The smallest sample size included in these analysis was the 168 patients taking nortriptyline with both serum and dose information available. Using G*Power (Faul et al. 2007), we calculated that in this sample, it is possible to detect an effect size explaining 4.7 % of the variance in outcome with 80 % power, at a *p* value threshold of 0.05. This corresponds to 0.52 points on the ASEC when measuring total ADR burden. When assessing study drop out, the power calculation for the survival model was conducted within STATA, using the drug-specific rate of study drop-out. In the nortriptyline-specific analysis, hazard ratios of 0.64 (or 1.56) could be detected at *p* < 0.05 with 80 % power. For the escitalopram-specific analysis, hazard ratios of 0.78 (or 1.28) could be detected.

## Results

Genotypic frequencies in this sample were typical of a Caucasian population (see Supplementary Materials for details), and P450 genotype was unrelated to age, sex, centre of recruitment or dose of antidepressant prescribed.

**Fig. 2:**
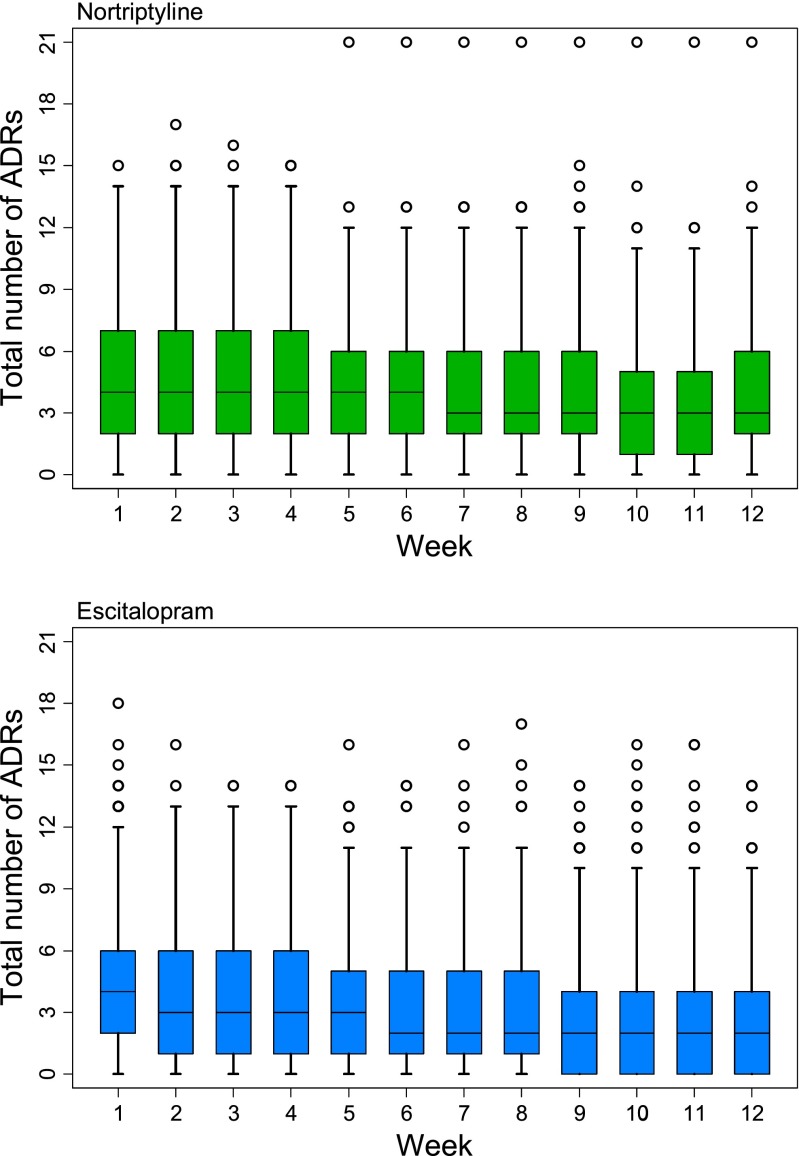
Total ADR burden per week, by drug. (*Box* outlines lower to upper quartiles with median markers, *whiskers* show 5th to 95th percentile, outliers are indicated by *hollow circles*)

Serum concentrations of antidepressant are shown in Table 1. Concentrations of drugs and primary metabolites were unrelated to age in this sample. Females had significant higher concentration of escitalopram (M = 31.56 μg/L, SD = 19.933) than males (M = 25.56 μg/L, SD = 16.44); *t*(273) = −2.47, *p* = 0.014. No significant differences were observed between males and females for desmethylcitalopram, nortriptyline and 10-hydroxynortriptyline concentrations. As expected, dose across the study was significantly related to serum levels of antidepressant measured at week 8 (see Supplementary Materials). There were also differences between centre of recruitment in terms of serum concentrations of antidepressant (see Supplementary Materials). Figure 2 shows the pattern of weekly overall ADR burden, whilst the frequency of each individual ADR is shown the Supplementary Materials.

**Table 1:** Measured serum concentrations (μg/L) of antidepressant drug and primary metabolite

Drug	Measure	Number	Mean	St Dev
Escitalopram	Escitalopram	275	29.60	19.04
	Desmethylcitalopram	205	11.25	5.21
Nortriptyline	Nortriptyline	184	93.35	59.28
	10-hydroxynortriptyline	180	67.99	53.37

Serum measurements were unavailable for some patients (as shown in Fig. 1). Amongst patients taking escitalopram with ADR ratings, total ADR burden was unrelated to whether serum measurements of antidepressant concentration were available or not. However, total ADR burden was associated with whether serum measurements were available amongst patients taking nortriptyline (*b* = 1.137, *t*(302) = 2.85, *p* = 0.005); higher ADR burden was seen in those patients without serum measurements.

### Overall ADR burden

The total number of ADRs was not predicted by P450 genotype. Given the previously observed association between P450 genotype and antidepressant tolerance when contrasts were made between PM vs all other genotypes (Mrazek et al. 2011), we also performed these comparisons in this sample, but still found no association for patients taking either nortriptyline or escitalopram. Additionally, serum concentrations of antidepressant did not predict total number of ADRs for either group. Results are shown in Table 2.

**Table 2:** CYP450 genotype and serum levels of antidepressant as predictors of overall ADR burden

Antidepressant	Predictor		Number	obs	*p* value	coeff	SE (robust)
Nortriptyline	*CYP2D6* genotype		251	2183	0.5638	−0.133	0.229
	*CYP2D6*; poor metabolisers vs all other genotypes		251	2183	0.5527	−0.321	0.541
	Serum concentration	nortriptyline	184	1890	0.8534	−0.029	0.154
		10-hydroxynortriptyline	180	1847	0.7323	0.063	0.183
		ratio (10-hydroxynortriptyline: nortriptyline)	178	1832	0.1895	0.232	0.176
		total (nortriptyline + 10-hydroxynortriptyline)	178	1832	0.7975	0.044	0.170
Escitalopram	*CYP2C19* genotype		340	3332	0.9627	−0.004	0.084
	*CYP2C19*; poor metabolisers vs all other genotypes		340	3332	0.5791	−0.361	0.649
	Serum concentration	escitalopram	275	2978	0.0852	0.250	0.145
		desmethylcitalopram	205	2242	0.1587	0.248	0.175
		ratio (desmethylcitalopram: escitalopram)	204	2230	0.6945	−0.066	0.167
		total (escitalopram + desmethylcitalopram)	204	2230	0.1155	0.260	0.165

### Specific ADRs

P450 genotype did not predict the presence of any specific ADRs, for patients taking either nortriptyline or escitalopram. There was also no significant association between serum concentrations of antidepressant and the majority of specific ADRs. However, for all patients (taking nortriptyline or escitalopram), a significant association was seen between dry mouth and serum concentrations of both drug and metabolite, as well as total concentration of antidepressant (Table 3).

**Table 3:** Dry mouth and serum concentration of antidepressant

(A) Primary analysis						
Antidepressant	Serum concentration	Number	obs	*p* value	OR	SE (robust)
Nortriptyline	**nortriptyline**	**184**	**1888**	**0.0023**	**1.826**	**0.362**
	**OH-nortriptyline**	**180**	**1845**	**1.20E-04**	**2.100**	**0.405**
	ratio (OH-nortriptyline/nortriptyline)	178	1830	0.0406	1.406	0.234
	**total** (**nortriptyline** + **OH-nortriptyline**)	**178**	**1830**	**4.97E-05**	**2.284**	**0.465**
Escitalopram	**escitalopram**	**275**	**2969**	**6.69E-04**	**1.480**	**0.170**
	**desmethylescitalopram**	**205**	**2233**	**0.0018**	**1.420**	**0.159**
	ratio (desmethylcitalopram/citalopram)	204	2221	0.6162	0.931	0.133
	**total** (**citalopram** + **desmethylcitalopram**)	**204**	**2221**	**0.0012**	**1.496**	**0.186**
(B) Secondary analysis; adjusting for effects of dose						
Antidepressant	Serum concentration	Number	obs	*p* value	OR	SE (robust)
Nortriptyline	nortriptyline	168	1698	0.0331	1.841	0.345
	**OH-nortriptyline**	**164**	**1664**	**0.0011**	**1.841**	**0.345**
	ratio (OH-nortriptyline/nortriptyline)	163	1652	0.0459	1.423	0.251
	**total** (**nortriptyline** + **OH-nortriptyline**)	**163**	**1652**	**0.0016**	**2.082**	**0.484**
Escitalopram	escitalopram	255	2715	0.0107	1.391	0.180
	desmethylescitalopram	190	2050	0.0189	1.326	0.159
	ratio (desmethylcitalopram/citalopram)	189	2038	0.9823	1.003	0.148
	total (citalopram + desmethylcitalopram)	189	2038	0.0319	1.358	0.194

Significant results in bold

Additionally, amongst patients taking escitalopram, significant associations were observed between diarrhoea and the ratio of metabolite to drug (*n* = 203, obs = 2210, *p* = 4.96 × 10^−4^, odds ratio (OR) = 0.597, SE = 0.088), as well as between dizziness and the concentration of metabolite (*n* = 202, obs = 2197, *p* = 3.28 × 10^−5^, OR = 1.564, SE = 0.168). Full results for all 21 ADRs tested are shown in the Supplementary Materials.

### Dose-adjusted analysis of significant associations

To probe the nature of the significant association between serum concentrations and these specific ADRs, we performed a secondary analysis, looking at the dose-independent relationships. When the effect of dose was removed, dry mouth was significantly associated with metabolite and total serum concentrations in patients taking nortriptyline (Table 3). The associations for diarrhoea with ratio of metabolite to drug (*n* = 188, obs = 2028, *p* = 0.0013, OR = 0.632, SE = 0.090) and dizziness with metabolite concentration (*n* = 188, obs = 2025, *p* = 1.05 × 10^−6^, OR = 1.658, SE = 0.172) in patients on escitalopram also remain significant when covarying for dose.

### Study discontinuation

P450 genotype was unrelated to study discontinuation, for patients taking either escitalopram (*n* = 376, hazard ratio = 0.870, *p* = 0.118), or nortriptyline (*n* = 284, hazard ratio = 1.300, *p* = 0.174). The (non-significant) hazard ratios given indicate the increase in relative risk of discontinuation, moving from less to more active P450 genotypes.

## Discussion

Cytochrome P450 genotype did not predict overall side effect burden, any of the 21 specific ADRs measured or study discontinuation in this sample. Further investigation, using serum concentration measures indicated that for both overall burden and the majority of specific ADRs, there was also no relationship with circulating levels of antidepressant. Exceptions to this general pattern were observed for dry mouth, dizziness and diarrhoea.

In the case of dry mouth, higher serum concentrations of antidepressant were linked to higher risk of the side effect for both escitalopram and nortriptyline. After adjusting for the influence of dose on serum concentration, the association with both metabolite and total serum concentration remained significant for patients taking nortriptyline. Amongst patients taking escitalopram, associations of diarrhoea with the ratio of metabolite to drug, and dizziness with concentration of metabolite were observed; both of these associations also remain significant after adjusting serum concentrations for prescribed dose of escitalopram. The absence of P450 genotypic association for each of these specific side effects indicates the importance of other factors that influence drug metabolism, beyond genetic variation in the cytochrome P450 enzymes.

When addressing the potential clinical impact for these associations with serum concentrations, it is important to consider baseline incidence of these specific ADRs; both diarrhoea and dizziness are rare side effects (with prevalence below 10 % of reports from patients taking escitalopram in this sample). This contrasts with dry mouth, which occurs in more than 70 % of reports from patients taking nortriptyline in this sample.

Despite the rarity of dizziness as a side effect to antidepressant treatment, it is nevertheless interesting to note that dizziness one of the few specific ADRS which shows significant association with treatment discontinuation in both the GENDEP (Uher et al. 2009a) and GenPod (Crawford et al. 2014) antidepressant treatment samples.

The failure to observe any association between P450 genotype and overall number of side effects holds even when collapsing genotype categories to compare poor metabolisers against all other genotypes; this is in contrast to a previous report examining tolerance to citalopram (Mrazek et al. 2011). However, power calculations suggest that the analyses presented here are sufficiently powered to detect any effects of a magnitude that would be clinically useful.

Furthermore, this study not only consider two different antidepressants (escitalopram and nortriptyline, which have divergent mechanisms of action), but also incorporate additional information on serum concentrations of antidepressant. This allows us to examine effects on drug metabolism beyond the genetic variability in P450 enzymes. However, even using this information, whilst three specific ADRs show evidence of association in drug-specific groups, we still fail to observe a link between rates of antidepressant metabolism and overall side effect burden for either escitalopram or nortriptyline.

The results presented here indicate that drug metabolism rates (and in particular P450 genotypes) are not of predictive of antidepressant side effects. But this should not be entirely surprising given what is known about the complexity of ADRs associated with antidepressant treatment. Pharmacokinetic factors beyond the cytochrome P450 enzymes (for example, the transportation of drug across the blood-brain barrier via P-glucoprotein 1), pharmacodynamic factors and the severity of depressive symptoms (Uher et al. 2009a) could all exert an influence on an individual’s likelihood of experiencing side effects with antidepressant treatment, indicating the antidepressant side effects are complex and multifactorial in nature. So, whilst drug levels may be linked to dry mouth, dizziness and diarrhoea, in order to accurately predict the majority of side effects, more complex and multifactorial modelling is required.

Nevertheless, all conclusions must be drawn within the context of the study design. In GENDEP, patients received treatment according to a flexible dosing protocol, where both treatment response and side effects could be used by clinicians to inform dose alterations throughout the study. This means that the doses received by patients were adjusted in response to treatment outcomes as the study proceeded. Thus, our conclusions indicate the extent to which data on P450 genotypes and serum concentrations of antidepressants are associated with the variation in ADRs and treatment discontinuation seen across 12 weeks, within a sample under clinical observation.

Therefore, we conclude that in this sample, where antidepressant dosage is monitored by clinicians and adjusted using their judgement, P450 genotypes are not predictive of treatment-associated side effects, as measured by overall side effect burden, specific ADRs or study discontinuation. However, there is some evidence that drug metabolism rates may play a role in the occurrence of dizziness and diarrhoea for patients taking escitalopram and of dry mouth for patients taking nortriptyline.

## Electronic supplementary material

Below is the link to the electronic supplementary material.

ESM 1 (DOC 515 kb)

## References

[CR1] Bull SA, Hunkeler EM, Lee JY, Rowland CR, Williamson TE, Schwab JR (2002). Discontinuing or switching selective serotonin-reuptake inhibitors. Ann Pharmacother.

[CR2] Couchman L (2012). Turbulent flow chromatography in bioanalysis: a review. Biomed. Chromatogr. : BMC.

[CR3] Crawford AA, Lewis S, Nutt D, Peters TJ, Cowen P, O’Donovan MC (2014). Adverse effects from antidepressant treatment: randomised controlled trial of 601 depressed individuals. Psychopharmacology (Berl).

[CR4] Dahl ML, Bertilsson L, Nordin C (1996). Steady-state plasma levels of nortriptyline and its 10-hydroxy metabolite: relationship to the CYP2D6 genotype. Psychopharmacology (Berl).

[CR5] de Leon J (2006). AmpliChip P450 test: personalized medicine has arrived in psychiatry. Expert Rev Mol Diagn.

[CR6] Faul F, Erdfelder E, Lang AG, Buchner A (2007). G*Power 3: a flexible statistical power analysis program for the social, behavioral, and biomedical sciences. Behav Res Methods.

[CR7] FDA (2001) Guidance for industry. Bioanalytical method validation. 2013

[CR8] FDA (2011) FDA: drug interactions (P450 inhibitors and inducers). 2013

[CR9] Freeman B, Smith N, Curtis C, Huckett L, Mill J, Craig IW (2003). DNA from buccal swabs recruited by mail: evaluation of storage effects on long-term stability and suitability for multiplex polymerase chain reaction genotyping. Behav Genet.

[CR10] Grasmäder K, Verwohlt P, Rietschel M, Dragicevic A, Müller M, Hiemke C (2004). Impact of polymorphisms of cytochrome-P450 isoenzymes 2C9, 2C19 and 2D6 on plasma concentrations and clinical effects of antidepressants in a naturalistic clinical setting. Eur J Clin Pharmacol.

[CR11] Hiemke C, Hartter S (2000). Pharmacokinetics of selective serotonin reuptake inhibitors. Pharmacol Ther.

[CR12] Hodgson K, Tansey K, Dernovsek MZ, Hauser J, Henigsberg N, Maier W (2014). Genetic differences in cytochrome P450 enzymes and antidepressant treatment response. J Psychopharmacol.

[CR13] Huezo-Diaz P, Perroud N, Spencer EP, Smith R, Sim S, Virding S (2012). CYP2C19 genotype predicts steady state escitalopram concentration in GENDEP. J Psychopharmacol (Oxford, England).

[CR14] Ingelman-Sundberg M (2004). Pharmacogenetics of cytochrome P450 and its applications in drug therapy: the past, present and future. Trends Pharmacol Sci.

[CR15] Kennedy S, McIntyre R, Fallu A, Lam R (2002) Pharmacotherapy to sustain the fully remitted state. Journal of Psychiatry and Neuroscience 27(4):269–280 PMC161661 12174736

[CR16] Kent JT (1982). Robust properties of likelihood ratio tests. Biometrika.

[CR17] Kirchheiner J, Brosen K, Dahl ML, Gram LF, Kasper S, Roots I (2001). CYP2D6 and CYP2C19 genotype-based dose recommendations for antidepressants: a first step towards subpopulation-specific dosages. Acta Psychiatr Scand.

[CR18] Kirchheiner J, Nickchen K, Bauer M, Wong ML, Licinio J, Roots I (2004). Pharmacogenetics of antidepressants and antipsychotics: the contribution of allelic variations to the phenotype of drug response. Mol Psychiatry.

[CR19] Linder MW, Keck PE (1998). Standards of laboratory practice: antidepressant drug monitoring. National Academy of Clinical Biochemistry. Clin Chem.

[CR20] Lingjaerde O, Ahlfors UG, Bech P, Dencker SJ, Elgen K (1987). The UKU side effect rating scale. A new comprehensive rating scale for psychotropic drugs and a cross-sectional study of side effects in neuroleptic-treated patients. Acta Psychiatr Scand Suppl 334:1–100 10.1111/j.1600-0447.1987.tb10566.x 2887090

[CR21] Masand PS (2003) Tolerability and adherence issues in antidepressant therapy. Clinical Therapeutics 25(8):2289–2304 10.1016/s0149-2918(03)80220-5 14512135

[CR22] Matchar DB, Thakur ME, Grossman I, McCrory DC, Orlando LA, Steffens DC et al. (2007) Testing for cytochrome P450 polymorphisms in adults with non-psychotic depression treated with selective serotonin reuptake inhibitors (SSRIs). Evid Rep Technol Assess (Full Rep), 2007/09/04 edn, pp 1–77 PMC4781099 17764209

[CR23] Montgomery SA, Asberg M (1979). A new depression scale designed to be sensitive to change. Br J Psychiatry.

[CR24] Mrazek DA, Biernacka JM, O’Kane DJ, Black JL, Cunningham JM, Drews MS (2011). CYP2C19 variation and citalopram response. Pharmacogenet Genomics.

[CR25] Murphy GM, Pollock BG, Kirshner MA, Pascoe N, Cheuk W, Mulsant BH (2001). CYP2D6 genotyping with oligonucleotide microarrays and nortriptyline concentrations in geriatric depression. Neuropsychopharmacology.

[CR26] Olesen OV, Linnet K (1999). Studies on the stereoselective metabolism of citalopram by human liver microsomes and cDNA-expressed cytochrome P450 enzymes. Pharmacology.

[CR27] Peters EJ, Slager SL, Kraft JB, Jenkins GD, Reinalda MS, McGrath PJ (2008). Pharmacokinetic genes do not influence response or tolerance to citalopram in the STAR D sample. PLoS One.

[CR28] Rebsamen MC, Desmeules J, Daali Y, Chiappe A, Diemand A, Rey C (2009). The AmpliChip P450 test: cytochrome P450 2D6 genotype assessment and phenotype prediction. Pharmacogenomics J.

[CR29] Reis M, Aamo T, Spigset O, Ahlner J (2009). Serum concentrations of antidepressant drugs in a naturalistic setting: compilation based on a large therapeutic drug monitoring database. Ther Drug Monit.

[CR30] Rudberg I, Mohebi B, Hermann M, Refsum H, Molden E (2008). Impact of the ultrarapid CYP2C19 17 allele on serum concentration of escitalopram in psychiatric patients. Clin Pharmacol Ther.

[CR31] Sim SC, Risinger C, Dahl ML, Aklillu E, Christensen M, Bertilsson L (2006). A common novel CYP2C19 gene variant causes ultrarapid drug metabolism relevant for the drug response to proton pump inhibitors and antidepressants. Clin Pharmacol Ther.

[CR32] Tansey KE, Guipponi M, Perroud N, Bondolfi G, Domenici E, Evans D (2012). Genetic predictors of response to serotonergic and noradrenergic antidepressants in major depressive disorder: a genome-wide analysis of individual-level data and a meta-analysis. PLoS Med.

[CR33] Trivedi MH, Rush AJ, Wisniewski SR, Nierenberg AA, Warden D, Ritz L (2006). Evaluation of outcomes with citalopram for depression using measurement-based care in STAR D: implications for clinical practice. Am J Psychiatry.

[CR34] Uher R, Farmer A, Henigsberg N, Rietschel M, Mors O, Maier W (2009). Adverse reactions to antidepressants. Br. J. Psychiatry: J Ment Sci.

[CR35] Uher R, Maier W, Hauser J, Marusic A, Schmael C, Mors O (2009). Differential efficacy of escitalopram and nortriptyline on dimensional measures of depression. Br J Psychiatry.

[CR36] Williams RL (2000). A note on robust variance estimation for cluster-correlated data. Biometrics.

[CR37] Wing JK, Sartorius N, Ostün TB (1998). Diagnosis and clinical measurement in psychiatry: a reference manual for SCAN.

