# Supplementary material for: Exploring the role of drug-metabolising enzymes in antidepressant side effects
Source: Psychopharmacology (Berl). 2015 Mar 12;232(14):2609–17. doi: 10.1007/s00213-015-3898-x (PMC4480333; doi:10.1007/s00213-015-3898-x)
Supplement: Supplementary file 1 — (DOC 515 kb) [file 213_2015_3898_MOESM1_ESM.doc]

**Supplementary Materials**

[ASEC 2](#__RefHeading___Toc377642002)

[CYP450 enzyme-inhibiting comedications 3](#__RefHeading___Toc377642003)

[CYP450 enzyme genotypic frequencies 4](#__RefHeading___Toc377642004)

[Dose and serum concentrations of antidepressant 5](#__RefHeading___Toc377642005)

[Centre of recruitment and serum concentrations of antidepressant 5](#__RefHeading___Toc377642006)

[Frequency of each ADR measured on the ASEC 6](#__RefHeading___Toc377642007)

[Association between specific ADRs and CYP450 genotype or serum levels of antidepressant. 14](#__RefHeading___Toc377642008)

**ASEC**

**Antidepressant Side-Effects Checklist**

Score the following list of symptoms (0-3; 0 = absent, 1 = mild, 2 = moderate, 3 = severe)

Please indicate if the symptom is *likely to be* a side effect of medication (either study drug, or another antidepressant). Write a comment to provide relevant information if the item is **not** a side effect.

| **List of symptoms** | | **Score (0-3)** | | | | **Linked to study drug?** | | **Comment** |
| --- | --- | --- | --- | --- | --- | --- | --- | --- |
| **1** | Dry mouth | 0 | 1 | 2 | 3 | Y | N |  |
| **2** | Drowsiness | 0 | 1 | 2 | 3 | Y | N |  |
| **3** | Insomnia (difficulty sleeping) | 0 | 1 | 2 | 3 | Y | N |  |
| **4** | Blurred vision | 0 | 1 | 2 | 3 | Y | N |  |
| **5** | Headache | 0 | 1 | 2 | 3 | Y | N |  |
| **6** | Constipation | 0 | 1 | 2 | 3 | Y | N |  |
| **7** | Diarrhoea | 0 | 1 | 2 | 3 | Y | N |  |
| **8** | Increased appetite | 0 | 1 | 2 | 3 | Y | N |  |
| **9** | Decreased appetite | 0 | 1 | 2 | 3 | Y | N |  |
| **10** | Nausea or Vomiting | 0 | 1 | 2 | 3 | Y | N |  |
| 1 = slight nausea, 2 = more nausea but no vomiting, 3 = with vomiting | | | | | | | |  |
| **11** | Problems with urination | 0 | 1 | 2 | 3 | Y | N |  |
| **12** | Problems with sexual function | 0 | 1 | 2 | 3 | Y | N |  |
| **13** | Palpitations | 0 | 1 | 2 | 3 | Y | N |  |
| **14** | Feeling light-headed on standing | 0 | 1 | 2 | 3 | Y | N |  |
| **15** | Feeling like the room is spinning around | 0 | 1 | 2 | 3 | Y | N |  |
| **16** | Sweating | 0 | 1 | 2 | 3 | Y | N |  |
| **17** | Increased body temperature | 0 | 1 | 2 | 3 | Y | N |  |
| **18** | Tremor | 0 | 1 | 2 | 3 | Y | N |  |
| **19** | Disorientation | 0 | 1 | 2 | 3 | Y | N |  |
| **20** | Yawning | 0 | 1 | 2 | 3 | Y | N |  |
| **21** | Weight gain | 0 | 1 | 2 | 3 | Y | N |  |
| Score: | | | | | | | |  |

**CYP450 enzyme-inhibiting comedications**

No patients were taking drugs associated with induction of CYP2C19 or CYP2D6.

Table S1: Number of patients prescribed escitalopram, with serum measurements available (N=266), also taking CYP450 inhibiting comedication.

| **Drug taken** | **CYP Enzymes Inhibited** | **Number of patients** |
| --- | --- | --- |
| Combined Oral Contraceptive Pill | CYP2D6 and CYP2C19 | 11 |
| Diltiazem | CYP2D6 | 1 |
| Propafenone | CYP2D6 | 1 |
| Verapamil | CYP2D6 | 1 |
| Omeprazole | CYP2C19 | 1 |

Table S2: Number of patients prescribed nortriptyline, with serum measurements available (N=191), also taking CYP2D6-inhibiting comedication. .

| **Drug taken** | **CYP Enzymes Inhibited** | **Number of patients** |
| --- | --- | --- |
| Combined Oral Contraceptive Pill | CYP2D6 and CYP2C19 | 8 |
| Amiodarone | CYP2D6 | 1* |
| Ranitidine | CYP2D6 | 1 |

*: this patient is also taking a COCP

**CYP450 enzyme genotypic frequencies**

Table S3: Categorisation of CYP2D6 alleles (as Rebsamen et al., 2009).

| Alleles tested | Categorisation of allelic activity level |
| --- | --- |
| *3/*4/*5/*6/*7/*15/*4XN | Non-functional |
| *9/*10/*14B/*17/*41/*41XN | Decreased function |
| *1/*2/*35 | Normal/wild type function |
| *1XN/*2XN/*35XN | Multiple copies of normal/wild type |

Table S4: CYP2D6 genotypic frequencies in patients taking nortriptyline

| Genotypic group | Classification criteria | Frequency in GENDEP | |
| --- | --- | --- | --- |
|  |  | N | % |
| PM | 2 non-functional alleles | 20 | 7.04 |
| IM | 1 non-functional, 1 decreased /  2 decreased alleles | 20 | 7.04 |
| EM | At least 1 wild type allele | 238 | 83.80 |
| UM | At least 3 copies of a wild type allele | 6 | 2.11 |

Table S5: CYP2D6 genotypic frequencies in patients taking escitalopram

| Genotypic group | Classification criteria | Frequency in GENDEP | |
| --- | --- | --- | --- |
|  |  | N | % |
| PM | 2 non-functional alleles | 28 | 7.59 |
| IM | 1 non-functional, 1 decreased /  2 decreased alleles | 34 | 9.21 |
| EM | At least 1 wild type allele | 290 | 78.59 |
| UM | At least 3 copies of a wild type allele | 17 | 4.61 |

Table S6: Categorisation of CYP2C19 alleles (as Mrazek et al., 2011)).

| Alleles tested | Categorisation of allelic activity level |
| --- | --- |
| *2 | Decreased function |
| *1 | Normal/wild type function |
| *17 | Increased function |

Table S7: CYP2C19 genotypic frequencies in patients taking escitalopram

| Genotypic group | Classification criteria | Frequency in GENDEP | |
| --- | --- | --- | --- |
|  |  | N | % |
| PM | *2/*2 | 7 | 1.86 |
| IM | *1/*2 | 74 | 19.68 |
| IM+ | *17/*2 | 30 | 7.98 |
| EM | *1/*1 | 144 | 38.30 |
| EM+ | *17/*1 | 97 | 25.80 |
| UM | *17/*17 | 24 | 6.38 |

**Dose and serum concentrations (µg/L) of antidepressant**

Weekly dose significantly predicts serum concentration of both drug and metabolite as measured at week 8, for both drugs.

| Serum measure | n | obs | p | Beta | Robust SE |
| --- | --- | --- | --- | --- | --- |
| nortriptyline | 168 | 1839 | 1.55x10^-6^ | 9.43 | 1.89 |
| 10-hydroxynortriptyline | 164 | 1805 | 7.24x10^-10^ | 11.28 | 1.72 |
| escitalopram | 255 | 2861 | 8.98x10^-15^ | 2.60 | 0.31 |
| desmethylcitalopram | 190 | 2155 | 7.33x10^-10^ | 2.98 | 0.46 |

**Centre of recruitment and serum concentrations (µg/L) of antidepressant**

All tests showed significant effects of centre of recruitment on serum concentrations (Nortriptyline; F(8, 175) = 5.74, p= 1.70x10^-06^; 10-Hydroxynortriptyline; F(8,171) = 8.01, p= 3.79 x 10^-9^; Escitalopram; F(8,266) = 7.07, p = 1.79 x10^-8^; Desmethylcitalopram; F(8,196) = 6.50, p=1.66 x10^-7^).

| Centre | Escitalopram | | | Desmethylcitalopram | | | Nortriptyline | | | 10-hydroxynortriptyline | | |
| --- | --- | --- | --- | --- | --- | --- | --- | --- | --- | --- | --- | --- |
|  | Mean | St Dev | n | Mean | St Dev | n | Mean | St Dev | n | Mean | St Dev | n |
| London | 37.61 | 19.71 | 29 | 9.56 | 2.99 | 18 | 82.70 | 49.24 | 20 | 71.66 | 44.42 | 20 |
| Brussels | 51.10 | 28.14 | 16 | 16.85 | 7.31 | 13 | 140.57 | 62.04 | 12 | 92.28 | 71.37 | 12 |
| Mannheim | 25.38 | 5.53 | 4 | 9.55 | 3.61 | 2 | 74.25 | 27.83 | 4 | 56.50 | 27.62 | 4 |
| Bonn | 27.84 | 14.94 | 44 | 11.17 | 3.59 | 39 | 113.83 | 51.83 | 35 | 98.51 | 55.59 | 35 |
| Brescia | 26.3 | 12.97 | 25 | 11.30 | 4.71 | 21 | 10.00 | 0 | 1 | 3.00 | 0 | 1 |
| Aarhus | 38.83 | 19.40 | 30 | 11.23 | 2.85 | 24 | 114.71 | 62.12 | 32 | 83.09 | 42.54 | 32 |
| Ljubljana | 25.17 | 16.22 | 31 | 9.58 | 2.69 | 24 | 72.40 | 57.25 | 26 | 58.39 | 54.22 | 25 |
| Poznan | 28.33 | 21.83 | 43 | 14.15 | 7.89 | 35 | 97.61 | 62.61 | 29 | 61.60 | 39.95 | 28 |
| Zagreb | 20.44 | 11.27 | 53 | 7.85 | 2.42 | 29 | 46.40 | 25.85 | 25 | 7.73 | 3.65 | 23 |

**Frequency of each ADR measured on the ASEC**

Each graph shows the percentage of patients reporting that ADR each week of the study, by drug. Black bars indicate patients taking nortriptyline whilst grey shows those on escitalopram.

**Association between specific ADRs and CYP450 genotype or serum levels of antidepressant.**

A) Patients taking nortriptyline

| ADR 1 | Dry mouth | n | obs | p | OR | SE |
| --- | --- | --- | --- | --- | --- | --- |
| CYP2D6 genotype | | 251 | 2182 | 0.111 | 0.710 | 0.153 |
| Serum concentration | nortriptyline | 184 | 1888 | 0.002 | 1.826 | 0.362 |
|  | 10-hydroxynortriptyline | 180 | 1845 | 1.20E-04 | 2.100 | 0.405 |
|  | ratio (10-hydroxynortriptyline: nortriptyline) | 178 | 1830 | 0.041 | 1.406 | 0.234 |
|  | total (nortriptyline + 10-hydroxynortriptyline) | 187 | 1830 | 4.97E-05 | 2.284 | 0.465 |
| ADR 2 | Drowsiness | n | obs | p | OR | SE |
| CYP2D6 genotype | | 251 | 2178 | 0.844 | 1.032 | 0.164 |
| Serum concentration | nortriptyline | 184 | 1884 | 0.695 | 1.054 | 0.142 |
|  | 10-hydroxynortriptyline | 180 | 1841 | 0.996 | 0.999 | 0.123 |
|  | ratio (10-hydroxynortriptyline: nortriptyline) | 178 | 1826 | 0.277 | 1.172 | 0.171 |
|  | total (nortriptyline + 10-hydroxynortriptyline) | 178 | 1826 | 0.794 | 1.033 | 0.129 |
| ADR 3 | Insomnia | n | obs | p | OR | SE |
| CYP2D6 genotype | | 251 | 2160 | 0.395 | 0.838 | 0.174 |
| Serum concentration | nortriptyline | 182 | 1866 | 0.486 | 0.908 | 0.126 |
|  | 10-hydroxynortriptyline | 179 | 1835 | 0.655 | 1.087 | 0.202 |
|  | ratio (10-hydroxynortriptyline: nortriptyline) | 177 | 1820 | 0.086 | 1.264 | 0.172 |
|  | total (nortriptyline + 10-hydroxynortriptyline) | 177 | 1820 | 0.921 | 0.985 | 0.153 |
| ADR 4 | Blurred vision | n | obs | p | OR | SE |
| CYP2D6 genotype | | 250 | 2172 | 0.681 | 1.125 | 0.323 |
| Serum concentration | nortriptyline | 183 | 1878 | 0.849 | 0.972 | 0.145 |
|  | 10-hydroxynortriptyline | 179 | 1835 | 0.514 | 0.916 | 0.123 |
|  | ratio (10-hydroxynortriptyline: nortriptyline) | 177 | 1820 | 0.775 | 1.040 | 0.142 |
|  | total (nortriptyline + 10-hydroxynortriptyline) | 177 | 1820 | 0.697 | 0.953 | 0.119 |
| ADR 5 | Headache | n | obs | p | OR | SE |
| CYP2D6 genotype | | 251 | 2176 | 0.127 | 1.320 | 0.241 |
| Serum concentration | nortriptyline | 183 | 1874 | 0.023 | 0.678 | 0.115 |
|  | 10-hydroxynortriptyline | 179 | 1831 | 0.325 | 0.857 | 0.134 |
|  | ratio (10-hydroxynortriptyline: nortriptyline) | 177 | 1816 | 0.220 | 1.165 | 0.145 |
|  | total (nortriptyline + 10-hydroxynortriptyline) | 177 | 1816 | 0.052 | 0.713 | 0.124 |
| ADR 6 | Constipation | n | obs | p | OR | SE |
| CYP2D6 genotype | | 251 | 2181 | 0.075 | 0.687 | 0.145 |
| Serum concentration | nortriptyline | 184 | 1887 | 0.175 | 1.224 | 0.183 |
|  | 10-hydroxynortriptyline | 180 | 1845 | 0.658 | 0.944 | 0.122 |
|  | ratio (10-hydroxynortriptyline: nortriptyline) | 178 | 1830 | 0.487 | 0.893 | 0.146 |
|  | total (nortriptyline + 10-hydroxynortriptyline) | 178 | 1830 | 0.472 | 1.097 | 0.141 |
| ADR 7 | Diarrhoea | n | obs | p | OR | SE |
| CYP2D6 genotype | | 251 | 2181 | 0.125 | 1.898 | 0.793 |
| Serum concentration | nortriptyline | 184 | 1888 | 0.654 | 1.113 | 0.266 |
|  | 10-hydroxynortriptyline | 180 | 1845 | 0.016 | 0.652 | 0.116 |
|  | ratio (10-hydroxynortriptyline: nortriptyline) | 178 | 1830 | 0.077 | 0.624 | 0.166 |
|  | total (nortriptyline + 10-hydroxynortriptyline) | 178 | 1830 | 0.579 | 0.879 | 0.204 |
| ADR 8 | Increased Appetite | n | obs | p | OR | SE |
| CYP2D6 genotype | | 251 | 2182 | 0.978 | 0.994 | 0.224 |
| Serum concentration | nortriptyline | 184 | 1888 | 0.191 | 1.212 | 0.178 |
|  | 10-hydroxynortriptyline | 180 | 1845 | 0.768 | 1.044 | 0.151 |
|  | ratio (10-hydroxynortriptyline: nortriptyline) | 178 | 1830 | 0.751 | 0.953 | 0.145 |
|  | total (nortriptyline + 10-hydroxynortriptyline) | 178 | 1830 | 0.248 | 1.174 | 0.163 |
| ADR 9 | Decreased Appetite | n | obs | p | OR | SE |
| CYP2D6 genotype | | 251 | 2180 | 0.696 | 0.923 | 0.189 |
| Serum concentration | nortriptyline | 184 | 1888 | 0.254 | 0.828 | 0.137 |
|  | 10-hydroxynortriptyline | 180 | 1845 | 0.451 | 0.871 | 0.160 |
|  | ratio (10-hydroxynortriptyline: nortriptyline) | 178 | 1830 | 0.662 | 1.086 | 0.204 |
|  | total (nortriptyline + 10-hydroxynortriptyline) | 178 | 1830 | 0.219 | 0.818 | 0.134 |
| ADR 10 | Nausea/vomiting | n | obs | p | OR | SE |
| CYP2D6 genotype | | 251 | 2176 | 0.740 | 0.911 | 0.257 |
| Serum concentration | nortriptyline | 184 | 1885 | 0.420 | 0.849 | 0.172 |
|  | 10-hydroxynortriptyline | 180 | 1842 | 0.122 | 0.763 | 0.133 |
|  | ratio (10-hydroxynortriptyline: nortriptyline) | 178 | 1827 | 0.577 | 1.090 | 0.169 |
|  | total (nortriptyline + 10-hydroxynortriptyline) | 178 | 1827 | 0.196 | 0.767 | 0.157 |
| ADR 11 | Problems with urination | n | obs | p | OR | SE |
| CYP2D6 genotype | | 251 | 2178 | 0.111 | 1.591 | 0.464 |
| Serum concentration | nortriptyline | 184 | 1885 | 0.318 | 0.704 | 0.248 |
|  | 10-hydroxynortriptyline | 180 | 1842 | 0.637 | 0.923 | 0.157 |
|  | ratio (10-hydroxynortriptyline: nortriptyline) | 178 | 1827 | 0.242 | 1.233 | 0.220 |
|  | total (nortriptyline + 10-hydroxynortriptyline) | 178 | 1827 | 0.477 | 0.846 | 0.199 |
| ADR 12 | Problems with sexual function | n | obs | p | OR | SE |
| CYP2D6 genotype | | 247 | 2087 | 0.814 | 0.960 | 0.166 |
| Serum concentration | nortriptyline | 181 | 1812 | 0.867 | 0.978 | 0.130 |
|  | 10-hydroxynortriptyline | 177 | 1769 | 0.969 | 0.994 | 0.150 |
|  | ratio (10-hydroxynortriptyline: nortriptyline) | 175 | 1754 | 0.760 | 0.954 | 0.148 |
|  | total (nortriptyline + 10-hydroxynortriptyline) | 175 | 1754 | 0.958 | 1.008 | 0.142 |
| ADR 13 | Palpitations | n | obs | p | OR | SE |
| CYP2D6 genotype | | 251 | 2178 | 0.933 | 0.985 | 0.182 |
| Serum concentration | nortriptyline | 184 | 1888 | 0.775 | 0.964 | 0.124 |
|  | 10-hydroxynortriptyline | 180 | 1845 | 0.567 | 1.069 | 0.125 |
|  | ratio (10-hydroxynortriptyline: nortriptyline) | 178 | 1830 | 0.096 | 1.249 | 0.167 |
|  | total (nortriptyline + 10-hydroxynortriptyline) | 178 | 1830 | 0.803 | 1.031 | 0.126 |
| ADR 14 | Feeling light-headed on standing | n | obs | p | OR | SE |
| CYP2D6 genotype | | 251 | 2180 | 0.867 | 1.033 | 0.199 |
| Serum concentration | nortriptyline | 184 | 1888 | 0.679 | 1.055 | 0.137 |
|  | 10-hydroxynortriptyline | 180 | 1845 | 0.178 | 1.187 | 0.151 |
|  | ratio (10-hydroxynortriptyline: nortriptyline) | 178 | 1830 | 0.425 | 1.106 | 0.139 |
|  | total (nortriptyline + 10-hydroxynortriptyline) | 178 | 1830 | 0.275 | 1.157 | 0.155 |
| ADR 15 | Feeling like the room is spinning | n | obs | p | OR | SE |
| CYP2D6 genotype | | 250 | 2167 | 0.184 | 1.343 | 0.298 |
| Serum concentration | nortriptyline | 183 | 1873 | 0.382 | 0.886 | 0.122 |
|  | 10-hydroxynortriptyline | 179 | 1830 | 0.848 | 1.030 | 0.159 |
|  | ratio (10-hydroxynortriptyline: nortriptyline) | 177 | 1815 | 0.533 | 1.105 | 0.177 |
|  | total (nortriptyline + 10-hydroxynortriptyline) | 177 | 1815 | 0.622 | 0.930 | 0.137 |
| ADR 16 | Sweating | n | obs | p | OR | SE |
| CYP2D6 genotype | | 251 | 2181 | 0.573 | 1.128 | 0.242 |
| Serum concentration | nortriptyline | 184 | 1887 | 0.028 | 0.720 | 0.108 |
|  | 10-hydroxynortriptyline | 180 | 1844 | 0.846 | 0.974 | 0.131 |
|  | ratio (10-hydroxynortriptyline: nortriptyline) | 178 | 1829 | 0.015 | 1.424 | 0.207 |
|  | total (nortriptyline + 10-hydroxynortriptyline) | 178 | 1829 | 0.130 | 0.812 | 0.112 |
| ADR 17 | Increased body temperature | n | obs | p | OR | SE |
| CYP2D6 genotype | | 248 | 2158 | 0.367 | 1.358 | 0.461 |
| Serum concentration | nortriptyline | 182 | 1867 | 0.007 | 0.399 | 0.136 |
|  | 10-hydroxynortriptyline | 178 | 1824 | 0.403 | 0.794 | 0.219 |
|  | ratio (10-hydroxynortriptyline: nortriptyline) | 176 | 1809 | 0.098 | 1.460 | 0.334 |
|  | total (nortriptyline + 10-hydroxynortriptyline) | 176 | 1809 | 0.021 | 0.514 | 0.148 |
| ADR 18 | Tremor | n | obs | p | OR | SE |
| CYP2D6 genotype | | 251 | 2181 | 0.022 | 0.641 | 0.124 |
| Serum concentration | nortriptyline | 184 | 1886 | 0.117 | 1.223 | 0.157 |
|  | 10-hydroxynortriptyline | 180 | 1843 | 0.627 | 1.061 | 0.130 |
|  | ratio (10-hydroxynortriptyline: nortriptyline) | 178 | 1828 | 0.660 | 1.065 | 0.154 |
|  | total (nortriptyline + 10-hydroxynortriptyline) | 178 | 1828 | 0.146 | 1.195 | 0.147 |
| ADR 19 | Disorientation | n | obs | p | OR | SE |
| CYP2D6 genotype | | 239 | 2070 | 0.643 | 1.139 | 0.321 |
| Serum concentration | nortriptyline | 183 | 1877 | 0.879 | 1.029 | 0.191 |
|  | 10-hydroxynortriptyline | 179 | 1834 | 0.604 | 0.923 | 0.143 |
|  | ratio (10-hydroxynortriptyline: nortriptyline) | 177 | 1819 | 0.837 | 0.965 | 0.166 |
|  | total (nortriptyline + 10-hydroxynortriptyline) | 177 | 1819 | 0.926 | 1.018 | 0.194 |
| ADR 20 | Yawning | n | obs | p | OR | SE |
| CYP2D6 genotype | | 251 | 2181 | 0.402 | 0.814 | 0.200 |
| Serum concentration | nortriptyline | 184 | 1887 | 0.642 | 0.922 | 0.161 |
|  | 10-hydroxynortriptyline | 180 | 1844 | 0.977 | 0.994 | 0.205 |
|  | ratio (10-hydroxynortriptyline: nortriptyline) | 178 | 1829 | 0.501 | 1.117 | 0.184 |
|  | total (nortriptyline + 10-hydroxynortriptyline) | 178 | 1829 | 0.708 | 0.930 | 0.181 |
| ADR 21 | Weight gain | n | obs | p | OR | SE |
| CYP2D6 genotype | | 251 | 2171 | 0.563 | 0.911 | 0.146 |
| Serum concentration | nortriptyline | 184 | 1876 | 0.361 | 1.123 | 0.143 |
|  | 10-hydroxynortriptyline | 180 | 1833 | 0.464 | 0.913 | 0.113 |
|  | ratio (10-hydroxynortriptyline: nortriptyline) | 178 | 1818 | 0.454 | 0.907 | 0.118 |
|  | total (nortriptyline + 10-hydroxynortriptyline) | 178 | 1818 | 0.772 | 1.038 | 0.133 |

B) Patients taking escitalopram

| ADR 1 | Dry mouth | n | obs | p | OR | SE |
| --- | --- | --- | --- | --- | --- | --- |
| *CYP2C19* genotype | | 340 | 3323 | 0.964 | 1.004 | 0.091 |
| Serum concentration | escitalopram | 275 | 2969 | 6.85E-04 | 1.480 | 0.170 |
|  | desmethylcitalopram | 205 | 2233 | 0.002 | 1.420 | 0.159 |
|  | ratio (desmethylcitalopram: escitalopram) | 204 | 2221 | 0.616 | 0.931 | 0.133 |
|  | total (escitalopram + desmethylcitalopram) | 204 | 2221 | 1.20E-03 | 1.496 | 0.186 |
| ADR 2 | Drowsiness | n | obs | p | OR | SE |
| *CYP2C19* genotype | | 336 | 3274 | 0.766 | 0.978 | 0.074 |
| Serum concentration | escitalopram | 272 | 2930 | 0.005 | 1.335 | 0.137 |
|  | desmethylcitalopram | 203 | 2206 | 0.003 | 1.314 | 0.122 |
|  | ratio (desmethylcitalopram: escitalopram) | 202 | 2194 | 0.293 | 0.872 | 0.114 |
|  | total (escitalopram + desmethylcitalopram) | 202 | 2194 | 0.007 | 1.357 | 0.153 |
| ADR 3 | Insomnia | n | obs | p | OR | SE |
| *CYP2C19* genotype | | 340 | 3323 | 0.936 | 1.006 | 0.071 |
| Serum concentration | escitalopram | 275 | 2968 | 0.434 | 0.919 | 0.099 |
|  | desmethylcitalopram | 205 | 2234 | 9.01E-01 | 1.014 | 0.117 |
|  | ratio (desmethylcitalopram: escitalopram) | 204 | 2222 | 0.499 | 1.081 | 0.125 |
|  | total (escitalopram + desmethylcitalopram) | 204 | 2222 | 0.497 | 0.922 | 0.110 |
| ADR 4 | Blurred vision | n | obs | p | OR | SE |
| *CYP2C19* genotype | | 339 | 3307 | 0.200 | 1.123 | 0.102 |
| Serum concentration | escitalopram | 275 | 2963 | 0.301 | 1.142 | 0.147 |
|  | desmethylcitalopram | 205 | 2227 | 0.077 | 1.230 | 0.144 |
|  | ratio (desmethylcitalopram: escitalopram) | 204 | 2215 | 0.381 | 1.125 | 0.152 |
|  | total (escitalopram + desmethylcitalopram) | 204 | 2215 | 0.237 | 1.193 | 0.179 |
| ADR 5 | Headache | n | obs | p | OR | SE |
| *CYP2C19* genotype | | 339 | 3311 | 0.770 | 0.979 | 0.070 |
| Serum concentration | escitalopram | 275 | 2966 | 0.983 | 1.002 | 0.096 |
|  | desmethylcitalopram | 205 | 2230 | 0.669 | 0.958 | 0.097 |
|  | ratio (desmethylcitalopram: escitalopram) | 204 | 2218 | 0.459 | 1.085 | 0.120 |
|  | total (escitalopram + desmethylcitalopram) | 204 | 2218 | 0.869 | 0.982 | 0.109 |
| ADR 6 | Constipation | n | obs | p | OR | SE |
| *CYP2C19* genotype | | 340 | 3322 | 0.214 | 0.868 | 0.099 |
| Serum concentration | escitalopram | 275 | 2967 | 0.477 | 1.107 | 0.159 |
|  | desmethylcitalopram | 205 | 2231 | 0.131 | 1.200 | 0.145 |
|  | ratio (desmethylcitalopram: escitalopram) | 204 | 2219 | 0.397 | 0.865 | 0.148 |
|  | total (escitalopram + desmethylcitalopram) | 204 | 2219 | 0.237 | 1.196 | 0.181 |
| ADR 7 | Diarrhoea | n | obs | p | OR | SE |
| *CYP2C19* genotype | | 339 | 3311 | 0.153 | 0.870 | 0.085 |
| Serum concentration | escitalopram | 274 | 2958 | 0.032 | 1.269 | 0.141 |
|  | desmethylcitalopram | 204 | 2222 | 0.608 | 1.066 | 0.133 |
|  | ratio (desmethylcitalopram: escitalopram) | 203 | 2210 | 4.96E-04 | 0.597 | 0.088 |
|  | total (escitalopram + desmethylcitalopram) | 203 | 2210 | 0.010 | 1.368 | 0.167 |
| ADR 8 | Increased Appetite | n | obs | p | OR | SE |
| *CYP2C19* genotype | | 338 | 3299 | 0.199 | 0.892 | 0.080 |
| Serum concentration | escitalopram | 272 | 2934 | 0.815 | 1.028 | 0.122 |
|  | desmethylcitalopram | 202 | 2199 | 0.522 | 0.886 | 0.168 |
|  | ratio (desmethylcitalopram: escitalopram) | 201 | 2187 | 0.316 | 0.835 | 0.150 |
|  | total (escitalopram + desmethylcitalopram) | 201 | 2187 | 0.537 | 0.905 | 0.147 |
| ADR 9 | Decreased Appetite | n | obs | p | OR | SE |
| *CYP2C19* genotype | | 340 | 3324 | 0.856 | 1.016 | 0.089 |
| Serum concentration | escitalopram | 274 | 2960 | 0.522 | 0.924 | 0.113 |
|  | desmethylcitalopram | 204 | 2224 | 0.949 | 0.990 | 0.151 |
|  | ratio (desmethylcitalopram: escitalopram) | 203 | 2212 | 0.544 | 1.083 | 0.143 |
|  | total (escitalopram + desmethylcitalopram) | 203 | 2212 | 0.940 | 0.989 | 0.141 |
| ADR 10 | Nausea/vomiting | n | obs | p | OR | SE |
| *CYP2C19* genotype | | 336 | 3276 | 0.637 | 1.041 | 0.089 |
| Serum concentration | escitalopram | 272 | 2932 | 0.452 | 1.100 | 0.140 |
|  | desmethylcitalopram | 202 | 2196 | 0.174 | 0.856 | 0.098 |
|  | ratio (desmethylcitalopram: escitalopram) | 201 | 2184 | 0.104 | 0.806 | 0.107 |
|  | total (escitalopram + desmethylcitalopram) | 201 | 2184 | 0.445 | 1.104 | 0.143 |
| ADR 11 | Problems with urination | n | obs | p | OR | SE |
| *CYP2C19* genotype | | 339 | 3304 | 0.977 | 1.004 | 0.130 |
| Serum concentration | escitalopram | 274 | 2949 | 0.697 | 1.078 | 0.208 |
|  | desmethylcitalopram | 204 | 2214 | 0.313 | 1.221 | 0.242 |
|  | ratio (desmethylcitalopram: escitalopram) | 203 | 2202 | 0.979 | 0.994 | 0.225 |
|  | total (escitalopram + desmethylcitalopram) | 203 | 2202 | 0.631 | 1.115 | 0.253 |
| ADR 12 | Problems with sexual function | n | obs | p | OR | SE |
| *CYP2C19* genotype | | 337 | 3220 | 0.664 | 1.035 | 0.082 |
| Serum concentration | escitalopram | 271 | 2866 | 0.200 | 1.176 | 0.149 |
|  | desmethylcitalopram | 202 | 2175 | 0.468 | 1.094 | 0.135 |
|  | ratio (desmethylcitalopram: escitalopram) | 201 | 2163 | 0.264 | 0.859 | 0.117 |
|  | total (escitalopram + desmethylcitalopram) | 201 | 2163 | 0.240 | 1.167 | 0.153 |
| ADR 13 | Palpitations | n | obs | p | OR | SE |
| *CYP2C19* genotype | | 339 | 3307 | 0.155 | 1.116 | 0.086 |
| Serum concentration | escitalopram | 274 | 2953 | 0.281 | 1.118 | 0.116 |
|  | desmethylcitalopram | 204 | 2217 | 0.008 | 1.311 | 0.133 |
|  | ratio (desmethylcitalopram: escitalopram) | 203 | 2205 | 0.278 | 1.129 | 0.126 |
|  | total (escitalopram + desmethylcitalopram) | 203 | 2205 | 0.298 | 1.137 | 0.140 |
| ADR 14 | Feeling light-headed on standing | n | obs | p | OR | SE |
| *CYP2C19* genotype | | 338 | 3298 | 0.120 | 1.193 | 0.135 |
| Serum concentration | escitalopram | 274 | 2957 | 0.978 | 1.004 | 0.145 |
|  | desmethylcitalopram | 204 | 2221 | 0.026 | 1.276 | 0.140 |
|  | ratio (desmethylcitalopram: escitalopram) | 203 | 2209 | 0.138 | 1.243 | 0.182 |
|  | total (escitalopram + desmethylcitalopram) | 203 | 2209 | 0.457 | 1.122 | 0.173 |
| ADR 15 | Feeling like the room is spinning | n | obs | p | OR | SE |
| *CYP2C19* genotype | | 338 | 3297 | 0.987 | 1.002 | 0.096 |
| Serum concentration | escitalopram | 272 | 2933 | 0.160 | 1.263 | 0.210 |
|  | desmethylcitalopram | 202 | 2197 | 3.28E-05 | 1.564 | 0.168 |
|  | ratio (desmethylcitalopram: escitalopram) | 201 | 2185 | 0.306 | 1.225 | 0.243 |
|  | total (escitalopram + desmethylcitalopram) | 201 | 2185 | 0.020 | 1.523 | 0.275 |
| ADR 16 | Sweating | n | obs | p | OR | SE |
| *CYP2C19* genotype | | 339 | 3311 | 0.553 | 1.047 | 0.082 |
| Serum concentration | escitalopram | 274 | 2957 | 0.474 | 1.069 | 0.100 |
|  | desmethylcitalopram | 204 | 2221 | 0.658 | 1.056 | 0.131 |
|  | ratio (desmethylcitalopram: escitalopram) | 203 | 2209 | 0.360 | 0.901 | 0.102 |
|  | total (escitalopram + desmethylcitalopram) | 203 | 2209 | 0.994 | 1.001 | 0.112 |
| ADR 17 | Increased body temperature | n | obs | p | OR | SE |
| *CYP2C19* genotype | | 339 | 3308 | 0.808 | 1.032 | 0.134 |
| Serum concentration | escitalopram | 273 | 2942 | 0.905 | 0.983 | 0.143 |
|  | desmethylcitalopram | 203 | 2206 | 0.106 | 0.723 | 0.146 |
|  | ratio (desmethylcitalopram: escitalopram) | 202 | 2194 | 0.136 | 0.744 | 0.147 |
|  | total (escitalopram + desmethylcitalopram) | 202 | 2194 | 0.131 | 0.805 | 0.116 |
| ADR 18 | Tremor | n | obs | p | OR | SE |
| *CYP2C19* genotype | | 338 | 3296 | 0.812 | 1.024 | 0.100 |
| Serum concentration | escitalopram | 273 | 2942 | 0.370 | 1.138 | 0.164 |
|  | desmethylcitalopram | 204 | 2219 | 0.146 | 1.215 | 0.163 |
|  | ratio (desmethylcitalopram: escitalopram) | 203 | 2207 | 0.971 | 1.005 | 0.143 |
|  | total (escitalopram + desmethylcitalopram) | 203 | 2207 | 0.180 | 1.234 | 0.194 |
| ADR 19 | Disorientation | n | obs | p | OR | SE |
| *CYP2C19* genotype | | 337 | 3286 | 0.893 | 0.984 | 0.115 |
| Serum concentration | escitalopram | 271 | 2919 | 0.540 | 1.088 | 0.150 |
|  | desmethylcitalopram | 202 | 2195 | 0.253 | 0.857 | 0.116 |
|  | ratio (desmethylcitalopram: escitalopram) | 201 | 2183 | 0.475 | 1.199 | 0.305 |
|  | total (escitalopram + desmethylcitalopram) | 201 | 2183 | 0.919 | 0.981 | 0.185 |
| ADR 20 | Yawning | n | obs | p | OR | SE |
| *CYP2C19* genotype | | 338 | 3300 | 0.366 | 1.088 | 0.101 |
| Serum concentration | escitalopram | 272 | 2935 | 0.154 | 1.163 | 0.123 |
|  | desmethylcitalopram | 202 | 2199 | 0.205 | 0.824 | 0.126 |
|  | ratio (desmethylcitalopram: escitalopram) | 201 | 2187 | 0.112 | 0.804 | 0.111 |
|  | total (escitalopram + desmethylcitalopram) | 201 | 2187 | 0.977 | 0.997 | 0.112 |
| ADR 21 | Weight gain | n | obs | p | OR | SE |
| *CYP2C19* genotype | | 338 | 3295 | 0.407 | 0.923 | 0.090 |
| Serum concentration | escitalopram | 273 | 2941 | 0.296 | 0.888 | 0.101 |
|  | desmethylcitalopram | 203 | 2206 | 0.364 | 0.878 | 0.126 |
|  | ratio (desmethylcitalopram: escitalopram) | 202 | 2194 | 0.571 | 0.921 | 0.134 |
|  | total (escitalopram + desmethylcitalopram) | 202 | 2194 | 0.132 | 0.819 | 0.108 |
